# Development of Epidermal Equivalent from Electrospun Synthetic Polymers for In Vitro Irritation/Corrosion Testing

**DOI:** 10.3390/nano10122528

**Published:** 2020-12-16

**Authors:** Denisse Esther Mallaupoma Camarena, Larissa Satiko Alcântara Sekimoto Matsuyama, Silvya Stuchi Maria-Engler, Luiz Henrique Catalani

**Affiliations:** 1 Laboratory of Polymeric Biomaterials, Department of Fundamental Chemistry, Institute of Chemistry, University of São Paulo, São Paulo 05508-000, Brazil; denisse23uni@usp.br; 2 Skin Biology Laboratory, Clinical Chemistry & Toxicology Department, School of Pharmaceutical Sciences, University of São Paulo, São Paulo 05508-000, Brazil; larissa.sekimoto@usp.br (L.S.A.S.M.); silvya@usp.br (S.S.M.-E.)

**Keywords:** keratinocytes, organotypic skin, epidermal equivalent, electrospinning

## Abstract

The development of products for topical applications requires analyses of their skin effects before they are destined for the market. At present, the ban on animal use in several tests makes the search for in vitro models (such as artificial skin) necessary to characterize the risks involved. In this work, tissue engineering concepts were used to manufacture collagen-free three-dimensional scaffolds for cell growth and proliferation. Two different human skin models—reconstructed human epidermis and full-thickness skin—were developed from electrospun scaffolds using synthetic polymers such as polyethylene terephthalate, polybutylene terephthalate, and nylon 6/6. After the construction of these models, their histology was analyzed by H&E staining and immunohistochemistry. The results revealed a reconstructed epidermal tissue, duly stratified, obtained from the nylon scaffold. In this model, the presence of proteins involved in the epidermis stratification process (cytokeratin 14, cytokeratin 10, involucrin, and loricrin) was confirmed by immunohistochemistry and Western blot analysis. The nylon reconstructed human epidermis model’s applicability was evaluated as a platform to perform irritation and corrosion tests. Our results demonstrated that this model is a promising platform to assess the potential of dermal irritation/corrosion of chemical products.

## 1. Introduction

The skin barrier is the first physical and chemical natural protection that shields our body from the external environment [1,2], and impairment of the skin barrier function can generate or aggravate skin diseases [3]. Skin is a tissue comprised of two primary sections, an epithelial compartment (epidermis) and a mesenchymal compartment (dermis) [2,4]. The epidermis is the outermost section of the skin. It is a complex epithelium that performs protective functions (barrier function), avoiding dehydration, excluding toxins, resisting mechanical stress, and participating in immune responses [5]. The epidermis is a structure with multiple layers of keratinocytes in different stages [6]. The second skin compartment is the dermis, which contains mainly fibroblasts among collagen bundles secreted by them. The dermal compartment’s principal function is to support the epidermis and provide skin elasticity [7].

Since the regulatory impositions in animals’ use began, several alternatives for in vitro evaluations have gained a more significant presence. The European Union prohibited animal testing of cosmetic products since 2013 [8,9], while in Brazil, this policy has been applied since 2019. Several approaches have been used to develop artificial skin models to overcome the need for animal testing, including the use of natural and synthetic matrices [7]. Skin models are also useful tools in the research of the skin’s functional mechanisms in the evaluation of transdermal drugs [10,11] and chemical testing in general. Many types of in vitro skin models have been developed to date, and some of these are commercially available. These skin tissues produced in vitro (human skin equivalents) can be classified into two types: full-thickness skin (FTS) and reconstructed human epidermis (RHE). The first consists of two layers, a first dermal layer composed of human fibroblasts embedded within the scaffolds, and a second layer comprised of keratinocytes seeded on top of the produced dermis. The RHE contains keratinocytes seeded directly onto the scaffold simulating a sole human epidermis, lacking a full dermal construction [12,13]. In vitro skin reconstructions can generate several model options; these variations can be justified using different technologies, protocols, or cell sources. [14]. Cytokeratins are the proteins that make up the cytoskeleton of epithelial cells and can be found with molecular weights (MW) between 40 to 70 kDa [15]. There are four major types of cytokeratins expressed in abundance on a healthy human epidermis (MW: 50, 56.5, 58, and 65–67 kDa) [15,16]. Cytokeratins are categorized in pairs according to their co-expression: (i) a pair synthesized by basal cells (molecular weights of 50 kDa/58 kDa), and (ii) another cytokeratin pair expressed by suprabasal cells (molecular weights of 56.5 kDa/65–67 kDa) [15]. During the proliferation of the basal keratinocytes, two main cytokeratins (KRT5 and KRT14) are synthesized and part of the epithelial cells’ cytoskeleton. Some keratinocytes in the basal stratum migrate and proliferate, generating the spinous layer, where a set of enzymes is synthesized after the loss of mitotic activity. Here the production of a new set of structural products characteristic of the cornification process begins [17]. The cytokeratins expressed by suprabasal cells are KRT1 and KRT10. These proteins are the first products synthesized in the cornification process, replacing the structures formed by KRT5 and KRT14 of the cytoskeleton. At advanced stages, the keratinocytes express other proteins (e.g., filaggrin) that keep changing the cytokeratin filaments. This event will promote the cytoskeleton’s rearrangement (cell shape) until the collapse of the keratinocytes. Concurrently, other structural proteins, including IVL (65 kDa) and loricrin (LOR; 26 kDa), are synthesized and crosslinked by enzymes (transglutaminases) to reinforce the cornified envelope [17,18].

To develop an in vitro skin model or human skin equivalents, tissue engineering concepts were applied by producing collagen-free three-dimensional scaffolds allowing cell growth and proliferation. Cell scaffolds were prepared by electrospinning, a technology that provides nano and microscale fibrous structures with interconnected pores. The produced non-woven mat mimics the architecture of natural extracellular matrices in tissues, helping cell infiltration [19,20], thus facilitating the formation of artificial functional tissue [21,22,23,24]. Although synthetic and natural polymers can be used in electrospinning [25,26], the first class possesses ample options for mechanical properties, easily attained experimental conditions, and is typically less expensive. The chosen synthetic polymers are classified as biostable, biocompatible, largely used in biomedicine [27,28,29], and with a degradation rate higher than 24 months [30,31,32,33].

This work describes the development and characterization of human skin equivalents (FTS and RHE) on electrospun scaffolds made from synthetic polymers, followed by the assessment of their applicability as a testing platform for skin irritation and corrosion, according to standards of the Organization for Economic Cooperation and Development (OECD).

## 2. Materials and Methods

### 2.1. Polymers and Solvents for Electrospinning

Poly(ethylene terephthalate) (PET) (M_v_~18 kg/mol, Product Number: 200255, Sigma-Aldrich, Milwaukee, WI, USA), poly(1,4-butylene terephthalate) (PBT) (M_v_~38 kg/mol, Product Number: 190942, Sigma-Aldrich, St. Louis, MO, USA), poly(N,N′-hexamethyleneadipinediamide) (N6/6) (Mn~35 kg/mol, Product Number: 429171, St. Louis, MO, Sigma-Aldrich), 1,1,1,3,3,3-hexafluoro-2-propanol (HFP) (≥99%, Product Number: 105228, St. Louis, MO, Sigma-Aldrich, USA), dichloromethane (DCM) (EMPARTA^®^ ACS, Product Number: 1.07020, Merck, Darmstadt, Germany), anhydrous chloroform (for HPLC, ≥99.9%, Product Number: 650498, Sigma-Aldrich, St. Louis, MO, USA), formic acid (FAc) (98–100%, UN-No. 1779, Merck, Darmstadt, Germany) and acetic acid (glacial, Product Number: 1.00063, Merck, Darmstadt, Germany).

### 2.2. Cell Culture

Fibroblasts and keratinocytes (human cells) were the cells studied. Both cells were isolated from donated foreskin samples from the University of São Paulo Hospital (Brazil). The human cells were isolated as previously described by Pennacchi et al. [34], under the approval of the local Ethics Committee (HU CEP Case No. 943/09, SISNEP CAAE 0062.0.1.98.000-9). Briefly, the foreskin was washed with phosphate-buffered saline (PBS) containing penicillin-streptomycin antibiotics (Pen-Strep; Gibco, Life Technologies, Carlsbad, CA, USA). Each donated sample was cleaned and fragmented before digestion with 4 mg/mL of dispase II (Roche, Life Sciences, Branford, CT, USA) overnight at 4 °C. Then, the dermis and epidermis were mechanically separated from each other. The dermis was subsequently incubated with 1 mg/mL of collagenase (Gibco, Life Technologies) for 6 h at room temperature to induce collagen fibroblasts’ release. The epidermis was incubated with 0.05% trypsin-EDTA for 15 min at 37 °C to promote cell dissociation. Fibroblasts removed from the dermis and keratinocytes removed from the epidermis were centrifuged at 1500× *g* for 3 min and seeded with specific culture media, as described below [34].

Normal human epidermal keratinocytes and fibroblasts were cultivated in a growth medium specific for each cell type and maintained in an incubator at 37 °C containing 7.5% and 5% CO_2_, respectively [13]. Keratinocytes cells were cultured in KGM Gold Keratinocyte Growth Medium BulletKit (KGM, Lonza, Walkersville, MD, USA) supplemented with isoproterenol 10^−6^ M (Sigma-Aldrich, St. Louis, MO, USA). Fibroblasts were cultured in Dulbecco’s Modified Eagle Medium (DMEM, Gibco, Life Technologies, Indianapolis, IN, USA) supplemented with 10% inactivated fetal bovine serum (FBS, Gibco, Life Technologies) and antibiotics (25 μg/mL ampicillin sodium salt and 100 μg/mL streptomycin, Gibco Life Technologies, Grand Carlsbad, CA, USA).

### 2.3. Construction of Human Epidermis Models

The electrospun polymer mats were cut into 11 mm diameter discs, sterilized with ethanol solution 70% for 20 min, and subsequently irradiated with germicidal UV-C (Dominant wavelength: 254 nm) for 10 min on each side. After that, the Solo polymeric discs were inserted in each well in a 24-well plate containing DMEM for 24 h for hydration. Subsequently, the polymeric discs were fixed in a porous structure to seed the cells [35].

The development of an epithelial model tissue was divided into two levels: (i) construction of a full skin equivalent and (ii) construction of human epidermis equivalent (without a dermis).

Skin equivalent—The protocol used for the skin equivalent construction onto electrospun mats was based on a modified USP-FTS model [13]. 1.5 × 10^5^ fibroblasts were seeded on the polymeric disc’s surface in a medium 9:1 DMEM with FBS and incubated at 37 °C in a 5% CO_2_ atmosphere for 24 h. After, 2.5 × 10^5^ keratinocytes were seeded on top of fibroblasts and co-cultured (37 °C, 5% CO_2_) through 24 h in a mixture 1:1 of KGM-Gold Bullet Kit medium with an in-house prepared culture medium (RAFT-skin, specific for skin model), described previously by Catarino et al. [13]. A transwell system and disc were submitted to an air–liquid interface for 11 days. The RAFT-skin medium is a mixture of DMEM, Ham’s-F12, FBS, and supplements (cholera toxin, insulin, apo-transferrin, hydrocortisone 21-hemisuccinate, epidermal growth factor) [13].

Epidermis equivalent—Human epidermal keratinocytes (2.5 × 10^5^ cells/disc) were seeded onto the polymer mat’s surface. The cells were submerged in a mixture 1:1 of KGM-Gold Bullet Kit medium with a RAFT-RHE medium [13,36] for 24 h, followed by 11 days at the air–liquid interface. The RAFT-RHE medium is a mixture of DMEM and HAM (3:1) with supplements (insulin, hydrocortisone, transferrin, cholera toxin, TGF-α, EGF) and 5% of conditional medium obtained from primary fibroblasts, as was described by Catarino et al. [13].

### 2.4. Test Substances

Chemical choices for skin corrosion and irritation assay protocols were determined based on OECD Guideline. The chemicals used were sodium dodecyl sulfate (SDS) (biotechnology grade, VWR Life Science, Solon, OH, USA), DL-lactic acid (Ph. Eur., Product Number: 69775, Fluka, Steinheim, Germany), glacial acetic acid (ACS, ISO, Reag. Ph. Eur., Product Number: 1.00063, Merck, Darmstadt, Germany), NaCl (EMSURE^®^, ACS, ISO, Reag. Ph. Eur, Product Number: 1.06404, Merck, Darmstadt, Germany) and KOH from Merck (Darmstadt, Germany). 3-(4,5-dimethykthiazol-2-yl)-2,5-diphenyltetrazolium bromide (MTT, 98%, Product Number: 135038, Sigma-Aldrich, St. Louis, MO, USA) and isopropyl alcohol (Labsynth, Diadema, SP, Brazil) were used for viability assay.

### 2.5. Polymer Scaffold Preparation and Characterization

Solutions for electrospinning were prepared in different proportions and solvent mixtures (Table 1). PET and PBT were solubilized in HFP:DCM, while N6/6 was dissolved in formic acid:chloroform mixture. All solutions were maintained under magnetic stirring at room temperature for 24 h (PET, PBT) or 48 h (N6/6). For each case, the electrospinning solutions were placed into a 10 mL syringe (HSW^®^ NORM-JECT^®^, Tuttlingen, Germany) coupled to a 20-gauge needle (7748-06-N720, HAMILTON) used in the electrospinning system with a syringe pump (PHD 2000 Infusion syringe pump, HARVARD Apparatus, Holliston, MA, USA) and a high voltage power source (Series EH, GLASSMAN High Voltage, Inc., High Bridge, NJ, USA). Table 2 shows the experimental conditions and process parameters. The polymer fibers were collected over a static collector.

Fiber diameters were evaluated by fitting to a normal Gaussian distribution with Kolmogorov–Smimov and Lilliefors test for normality, using Statistica 12 software (StatSoft, Round Rock, TX, USA). The *p*-value values less than 0.05 (Lilliefors values) were obtained, which means that the hypothesis of being normal is rejected. Data are represented by box diagrams to evaluate the mean and the interquartile range (IQR) using GraphPad Prism 8 software.

### 2.6. Quality Control of the Tissue Model

Our products have the quality control of USP-FTS or USP-RHE models (according to each case) [13].

#### 2.6.1. Histology and Immunohistochemistry

After incubation, the samples were washed with PBS and fixed by immersion in 10% (*v*/*v*) neutral buffered formaldehyde for 4 h at 4 °C [37,38,39,40]. Subsequently, they were dehydrated (by ethanol and xylene) and embedded in paraffin. For each case, histological sections with 3 µm thickness were obtained with a semi-motorized rotary Leica RM2245 microtome (Leica Biosystems Inc., Buffalo Grove, IL, USA). The slides were deparaffinized and hydrated, dipped in xylene, ethanol (concentration of 100%, 95%, 80%, 70%, and 50%), and rinsed in water. Then, the sections were ready for histological or immunohistochemistry treatment. For the histological analysis, the hydrated sections were subjected to staining with hematoxylin-eosin (H&E). For immunohistochemistry analysis, antigen recovery was performed twice in Tris/EDTA buffer, pH 9 for 5 min at 95 °C. Immunolabeling assay was carried out using a mouse monoclonal antibody [LL002] to cytokeratin 14 (Abcam-7800), dilution 1:200; rabbit monoclonal antibody [EP 1607IHCY] to cytokeratin 10 (Abcam 76318), dilution 1:150; rabbit polyclonal antibody to loricrin (Abcam-85679), dilution 1:100 and rabbit polyclonal antibody to involucrin (Abcam-27495), dilution (1:1000). A commercial kit with goat secondary antibody against rabbit and mouse immunoglobulins (EnVision Flex/HRP, Dako Omnis, Santa Clara, CA, USA) was used in combination with 3,3′-diaminobenzidine (DAB; EnVision Flex DAB + Chromogen, Dako Omnis, Santa Clara, CA, USA) according to the manufacturer’s instructions. All images were observed under AxiosKop 40 Carl Zeiss and photographed with a Zeiss Axiocam MRc5 camera using the Axiovission program, version 4.8 (Zeiss, Oberkochen, Germany), for image acquisition. All sections were compared with the control USP-RHE or USP-FTS model [13].

#### 2.6.2. Protein Expression by Western Blotting

The cells from RHEs tissues were lysed, according to Hieda et al. [41]. Protein concentrations were determined with the Pierce BCA protein assay kit (Thermo Fisher Scientific, USA). Total protein (40 µg) was subjected to electrophoresis in 8%, 12%, and 15% polyacrylamide gels under reducing conditions and transferred to polyvinylidene fluoride membranes (Hybond, Amersham Pharmacia Biotech, Piscataway, NJ, USA). The membranes were blocked in 3% BSA diluted in TBS-Tween 20 [41] for 1 h at room temperature and probed with the following antibodies: against cytokeratin 14 (Abcam-ab7800 1:1000), against cytokeratin 10 (Abcam ab76318 1:1000) and against involucrin (Abcam-ab27495 1:1000), and against β-actin (Abcam ab8227 1:2000) overnight at 4 °C. After washing, the membranes were incubated for 1 h at room temperature with the secondary antibody. Protein bands were detected by an enhanced chemiluminescence system (ECL, Amersham Pharmacia Biotech, Piscataway, NJ, USA).

#### 2.6.3. Cell Viability Measurement by MTT

Cell viability assay was determined by measuring the cellular metabolic activity using MTT [42,43,44], and performed according to OECD (OECD TG 431 and OECD TG 439) [45,46]. After the incubation time, the samples were washed before being exposed to any substance. The epidermis was exposed to a negative control solution, washed with PBS, and incubated with MTT (1 mg/mL) for 3 h (37 °C in 5% CO_2_). Then, the epidermis was washed with PBS to eliminate unreacted MTT. The reduced formazan was extracted by shake with 2 mL of isopropanol for 2 h. The OD was measured at 570 nm [45,47] using a plate reader (BioTek-Synergy HT., Winooski, VT, USA). The epidermis was exposed to different chemicals (test substance) and negative control. From the *OD* readings, the relative viability (*RV*) was calculated according to Equation (1) [13,47]:

(1) $$RV\left(\%\right)=\frac{{\left[OD\right]}_{test\;substance}}{{\left[OD\right]}_{control\;}}\times 100$$

### 2.7. Applications of the Epidermis

According to OECD protocols, the applicability of these dermal models was tested for two types of in vitro applications: testing for corrosion and irritation capacity. MTT assays and histological analyses were performed in two independent experiments (n = 2), and each tested in three tissue replicates. MTT results were statistically analyzed by the GraphPad Prism 8 software (GraphPad Software, Inc., San Diego, CA, USA).

#### 2.7.1. Corrosion Test

The applicability of this epidermal model for skin corrosion was evaluated based on OECD TG 431. From the substances listed on test guideline 431, three were chosen: NaCl (0.9%) (negative control), glacial acetic (positive control), and lactic acid (testing substance). From this assay, a material is considered corrosive when the RV value is lower or equal to 50%. These three substances were applied topically on the epidermal model’s surface after 11 days of incubation under the air–liquid interface. First, each model was transferred to a new plate (12-well) containing 300 µL new media. Then, 50 µL of substances (corrosive or non-corrosive) were applied to the epidermis surface and maintained incubated for 60 min (37 °C, 5% CO_2_). One epidermis sample was used for histological analysis of each substance, and a second sample was used for RV analysis by MTT assay.

#### 2.7.2. Irritation Test

The irritation testing applicability of the dermal model was accessed by the OECD TG 439 protocol with modifications. The surface of epidermal models was exposed to the following substances [45]: PBS (negative control), SDS 5% (w/v) (positive control), and KOH 5% (testing substance). According to OECD protocols, if RV ≤ 50%, the test substance is classified as a skin irritant. After the 11 days incubation period, the samples were transferred to a new plate (12-well) containing 300 µL fresh media. To each epidermal surface, 30 µL of the testing solution was applied and maintained for 20 min to room temperature. Following, each tissue was rinsed with PBS and transferred to a new plate (6-well) containing 1 mL fresh media, and subsequently maintained in the incubator (37 °C, 5% CO_2_) for 42 h. Then, each substance was removed from the surface by washing with PBS before any characterization. For each substance, one epidermal tissue was used for histological analysis, and a second was used to determine the RV values.

## 3. Results and Discussion

In this work, we show a model based on the collagen’s exemption while using inexpensive commodity plastics. The use of electrospinning as a processing technique results in great flexibility and readiness, properties that can facilitate production and increase its availability. We believe that avoiding the use of animal protein, we increase the predictive capacity of the model in response to a human tissue model. Collagen is a decellularized material, which brings source (animal donor) and method (purification) variability. Conversely, synthetic polymers are highly reproducible and clean materials.

This work is divided into three main objectives: (i) production of suitable polymeric mats to be used as cell scaffolds, (ii) construction and characterization of the tissue model, and (iii) testing its applicability. The assembly of such organotypic cultures depends on the fabrication of a viable polymeric scaffold that guarantees the tissue model’s quality. Furthermore, the relevance of the model was validated by its evaluation as a platform to perform irritation and corrosion tests.

### 3.1. Fabrication of Electrospun Polymer Scaffolds

Three different synthetic polymers were chosen to challenge the optimal characteristics of collagen surfaces typically used in epithelial models. The polymer mat must address the classic biomimetic needs for cell migration, attachment, and proliferation. Moreover, it must add advantages as convenient processability and handling, longer shelf-life, facile production, and low cost. Aiming at these characteristics, PET, PBT, and N6/6 were chosen as possible candidates.

The composition and concentration of each polymer’s solution were experimentally selected to ensure spinning stability and mat uniformity (Table 1 and Figures S1 and S2). SEM was used to characterize the surface topography, morphology, and size of the electrospun fibers. Figures S1 and S2 show images from the conditions that generated bead-free mats (green box).

**Figure 1:**
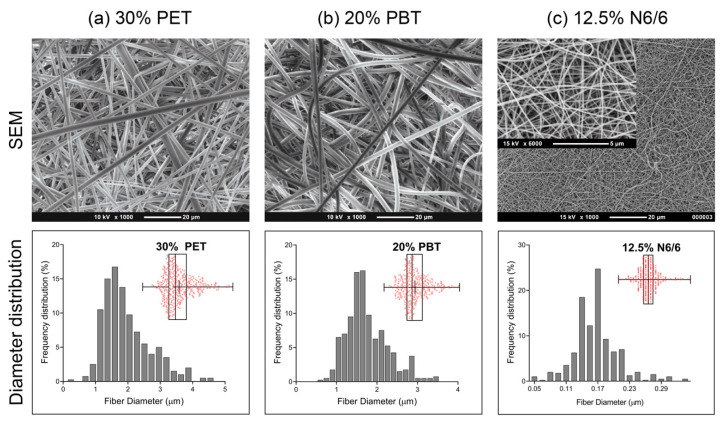
SEM micrographs and fiber frequency distribution of electrospun polymer mat surfaces. These images represent the characteristics of the meshes used in in vitro experiments. Full box plot analysis of (**a**–**c**) are shown in Figures S5–S7. Diameters were measured from eight SEM micrographs and analyzed from eight different locations on each mat (approximately 15 × 15 cm). A total of 400 measurements were taken (50 measurements from each image).

Fiber diameter histograms were evaluated and presented in Figures S3–S6. From these results, it is possible to indicate as optimal conditions for the use of PET as 30% (w/v) concentration using HPF:DCM (7:3) or HFP:CHCl_3_ (7:3) as the solvent. The average diameters did not show a significant difference; however, the condition which used the lowest proportion of HFP solvent was used. By applying the same approach for the PBT, ratios of 20% (w/v) concentration in HPF:DCM (7:3) or HFP:CHCl_3_ (7:3) as a solvent were chosen. For N6/6 mats, the concentration of 12.5% (w/v) in a mixture of FAc:CHCl_3_ (7.5:2.5) was found to be optimal. Figure 1 shows the SEM images, data density (by histogram), and dispersion of the data (by boxplot) of fiber diameters in each electrospun mat of PET, PBT, and N6/6. The distributions have unimodal type characteristic, and the mean diameter of the fibers was determined as 1.9 ± 0.7 µm for PET, 1.7 ± 0.5 µm for PBT, and 0.13 ± 0.03 µm for N6/6 (Figures S5–S7). The order of dispersion of the data can be established from the IQR values (Figures S5–S7) as N6/6 < PBT < PET.

### 3.2. Construction and Characterization of the Epidermal Model

One of the aims of this study was to build three-dimensional epidermal models. These models are known in the literature as human skin equivalents (SEs) [48]. SEs may either be FTS or RHE. FTS model is made up of the dermis (presence of fibroblasts) and epidermis (comprising the stratum corneum and viable keratinocytes) [48,49], whereas the RHE model exclusively contains the epidermis [9,50]. The importance of SEs is that they mimic native skin’s cellular organization, differentiation, function, composition [48,51], becoming a valuable option to assess the risks of topical products [52].

It is known from the literature that the physical characteristics of scaffolds can influence cell morphology, migration, and cell differentiation [21]; for example, the fiber orientation can influence cell viability and proliferation, affecting cell orientation in tissue development [53]. However, after the construction of the tissues, their morphology was evaluated by histology with H&E staining. To compare the FTS and RHE models with real skin morphology, human skin biopsies were previously obtained and thoroughly analyzed (Figure S8) [36]. The morphological parameter is a critical quality control described in the OECD Guidelines (TG439) [45].

#### 3.2.1. FTS Production

Fibroblasts and keratinocytes were seeded onto the built scaffolds (Section 2.3) to assess these polymers’ influence in the formation of FTS models. After 11 days of incubation of keratinocytes in the scaffold with fibroblast, the generation of epithelial tissue onto the polymer scaffolds used was demonstrated.

**Figure 2:**
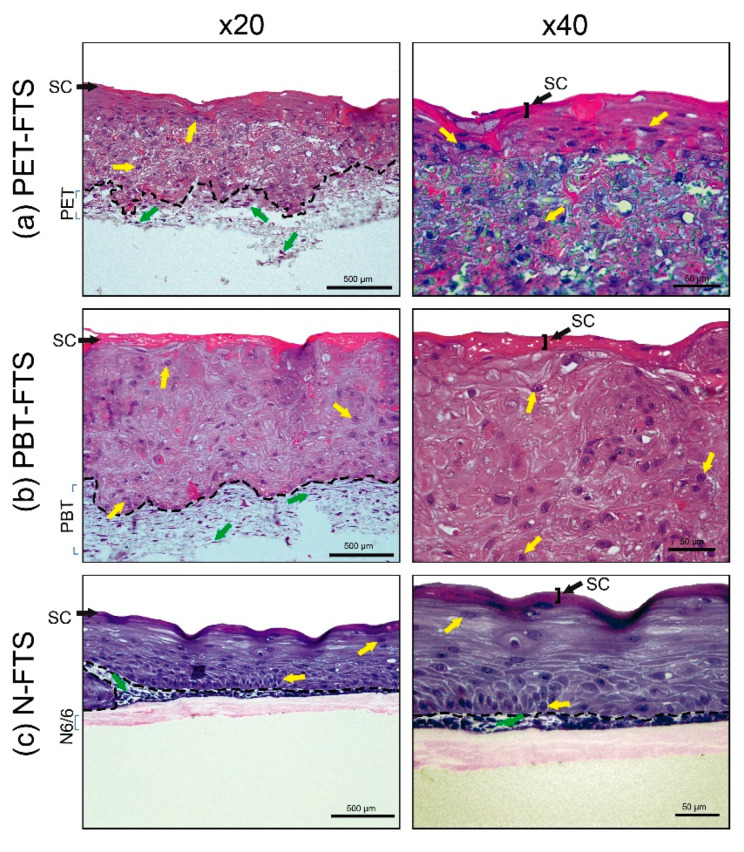
Fibroblasts (shown by green arrows) and keratinocytes (shown by yellow arrows) seeded on (**a**) PET, (**b**) PBT, and (**c**) N 6/6 scaffolds. The histology of the polymers-full-thickness skin (FTS) shows a quite delimited dermis and epidermis (shown by black dotted lines). SC: Stratum corneum (shown by black arrows). Magnification = 20×, bar = 500 µm. Magnification 40×, bar = 50 µm.

**Figure 3:**
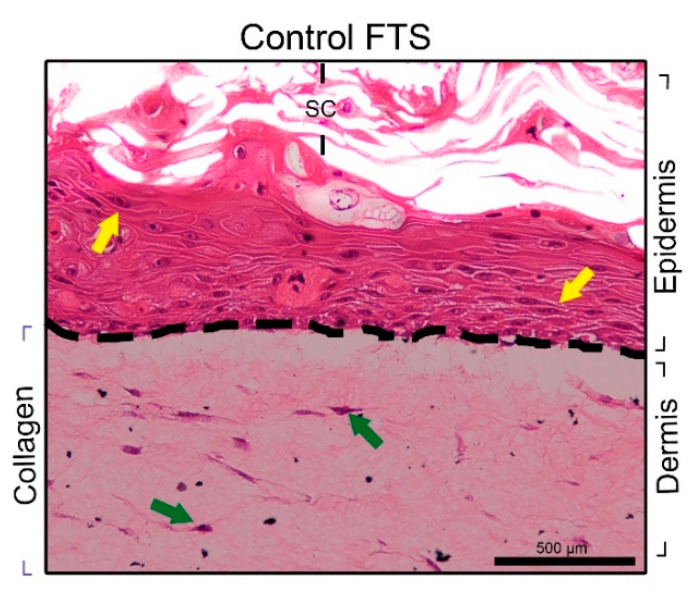
Photomicrographs of histological analysis—H&E staining (sample in paraffin) of the control FTS (USP-FTS). Fibroblasts (shown by green arrows) and keratinocytes (shown by yellow arrows) seeded on collagen. The histology of the control had shown quite delimited dermis and epidermis (shown by black dotted lines). SC: stratum corneum. Bar = 500 µm.

The images obtained after staining with H&E are shown in Figure 2 and Figure 3. The constructed FTSs on the polymeric scaffolds were named according to the polymer of the scaffold: PET-FTS (FTS using scaffold of PET), PBT-FTS (FTS using scaffold of PBT), N-FTS (FTS using scaffold of N6/6). Histological examination revealed that cell reconstructions on polymers (PET, PBT, and N6/6) always show quite delimited dermis and epidermis (black dotted lines), with the presence of fibroblasts (green arrows) and keratinocytes (yellow arrows) respectively (Figure 2). In the epidermal regions, the presence of stratum corneum is observed.

The morphological analysis reveals the interaction between cells and scaffolds, indicating the existence of adhesion and proliferation of fibroblasts in the scaffolds of PET and PBT (Figure 2) in the regions, which, by their morphologies, represent the dermis for each tissue. The fibroblast permeation was not observed in nylon mats (Figure 2c), most likely due to the smaller pore size of the mat, as can be seen in SEM images (Figure 1) when compared with the PET and PBT mats.

The USP-FTS [13,34] model was constructed as a quality reference, which we considered the control for the FTSs build (Figure 3). The keratinocytes cells did not show a proper stratification in the generation of the epidermis only in the PBT-FTS (Figure 2b). However, PET-FTS (Figure 2a) and N-FTS (Figure 2c) showed organized stratification in the epidermal region. Conversely, PET-FTS images were not optimal due to PET mats were damaged in histological processing due to their grain of solubility with some solvents, which interfere in the interpretation of images.

The N-FTS is presented as a promising model. Still, to overcome the inhomogeneous distribution of proliferated fibroblasts on the surface of the nylon scaffold, the construction of the RHE model was proposed (following item).

#### 3.2.2. RHE Production

**Figure 4:**
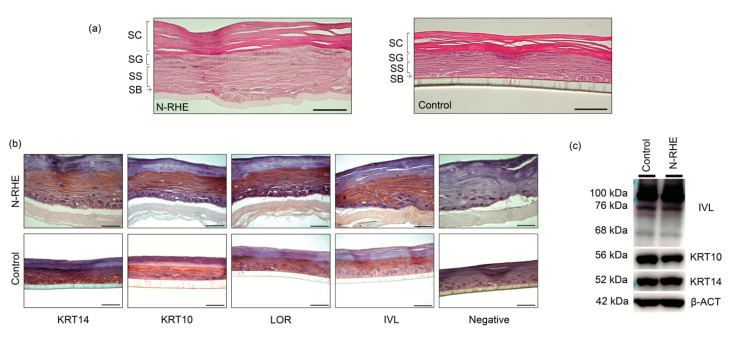
Morphology and immunohistochemistry of reconstructed human epidermis. Reconstructed human epidermis (RHEs) where N-RHE is the model with an N6/6 scaffold and the control model is the USP-RHE model. (**a**) Comparison between the control (USP-RHE) model and N-RHE model by H&E staining: Stratum corneum (SC), stratum granulosum (SG), stratum spinosum (SS), stratum basale (SB); (**b**) immunostaining of N-RHE and control models with KRT10, KRT14, LOR, IVL expression and negative (free of primary antibody); (**c**) Western blotting analysis for KRT10, KRT14, IVL, β-ACT. Magnification = 50×. Bar = 50 µm.

RHE is a morphologically well-differentiated epidermis characterized by the presence of stratified keratinocytes. Figure 4a right shows the control built by the USP-RHE protocol. The typical structure formed by strata corneum, granulosum, spinosum, and basale can be clearly seen. The new N-RHE model was developed from the evidence of well-formed keratinocytes stratifications, as mentioned previously, using nylon mats (Section 3.2.1). Figure 4a left shows histological images depicting the proper stratification compared to the control.

Keratinocytes present in the skin are responsible for constructing a protective barrier, separating the organism from the external environment. This barrier is formed by two distinct structures where the keratinocytes are assembled: a system with filaments composed of cytokeratin (KRT) and another structure from involucrin (IVL) with other proteins. This process of producing structural components in the epidermal barrier is part of the differentiation process [16]. In epidermal differentiation, keratinocytes in the basal layer migrate, proliferate, and differentiate until they generate the cornified layer. All of these steps are sequential and specific proteins are expressed in each one [17].

The epidermal models’ stratification features were evaluated by specific protein detection expressed by the keratinocytes in the epidermal differentiation process by using immunohistochemistry.

Figure 4b shows images obtained by the immunostaining of four different proteins. The immunohistochemical characterization of RHEs cultured for 11 days at the air–liquid interface showed the expression of two proteins: KRT14, which is characteristic in the basal keratinocytes (stratum basale), and KRT10 expressed in the suprabasal layer. Immunohistochemical staining also revealed the expression of LOR and IVL (stratum granulosum and upper stratum spinosum, respectively); these terminal differentiation markers confirmed the success of the cornification process of the epidermal model. The antibody-KTR14 used in this research marks the stratum basale, spinosum, and granulosum, but it does not mark the stratum corneum [54,55]. KRT10 is not synthesized in the stratum basale, but it was used to show that the produced KRT14 is expressed mainly in the stratum basale. The negative controls consisted of non-treated cells with primary antibodies; cells were stained with hematoxylin only. The human epidermis is a complex structure because it is composed of stratified tissue. In the process of stratification, keratinocytes are subjected to cytoarchitectural changes from the stratum basal to when they are keratinized (cornification). In this research, the epidermal stratification has been replicated in vitro assays. The RHEs models are constituted by different layers showing similarities with the four main layers of the native human epidermis (basal, spinous, granular, and cornified). The expression of biomarkers confirmed the presence of proteins from the different stages of the epidermis. It is important to note that our models satisfactorily represent the characteristics of the human epidermis, remembering that N-RHE models do not require the use of collagen as the scaffold [56]. Our results in the N-RHE model are in agreement with those demonstrated by Pedrosa et al. [36] in the morphological characterization in the RHE product (Figure S8) and also with those shown by Hieda et al. [41] in the immunohistochemical characterization of the RHE model.

Western blotting was carried out to corroborate the presence of proteins produced in the epidermal stratification [57]. This analysis confirmed protein expression in RHEs (Figure 4C and Figure S9) and revealed bands reported in the literature. The bands with MW~52 kDa [58,59,60,61] relative to the expression of KRT14, typical of proliferative keratinocytes, ~56 kDa [58,61,62,63] reveals the expression of KRT10 present in the spinous stratum, and the bands of IVL are 68 kDa [6,41], ~76 kDa [64], 120–140 kDa [58,60,61]. The size of the IVL is 68 kDa [6,41], and the additional bands are the characteristic of a larger structure due to the interaction of the involucrin with other molecules in the differentiation process, as shown by other authors, of ~76 kDa [64] and 120–140 kDa [58,60,61]. Involucrin is a precursor protein of the corneocyte layer; however, it is generated under the cell membrane’s inner surface (in keratinocytes) in the process of differentiation and production of the stratum corneum. IVL appears for the first time in the cell cytoplasm, and then it is crosslinked by transglutaminase with other proteins, generating larger structures [6,17]. However, it is not odd to obtain bands of a larger structure corresponding to a specific antibody against IVL.

### 3.3. Applications of N-RHE

There are several in vitro commercial models that are used to assess skin damage (EpiSkinTM, EpiDermTM, SkinEthicTM RHE, epiCS^®^, LabCyte EPI-MODEL24 SCT), produced on scaffolds of different materials, e.g., inert polycarbonate filters, polycarbonate with a coat of collagen [65]. The typical chemical substances to which these skin models are tested against are those with corrosive and/or irritant action. As described by United Nations (UN) Globally Harmonized System of Classification and Labelling of Chemicals (GHS), skin corrosion refers to irreversible damage after exposure to chemicals substances, manifested as visible necrosis through the epidermis and into the dermis [46], while skin irritation refers to reversible damage [45]. In this section, the N-RHE model was exposed to corrosive/irritant substances, and the potential damage was evaluated.

Similar to most commercial models available, the presented N-RHE model is limited in mimicking authentic skin due to the lack of appendages, as hair structures and glands. We trust that this model is robust and flexible enough to support future modifications to draw closer to human skin. The objective here is to introduce a new inexpensive and reliable protocol, committed to the OECD guidelines. Nevertheless, a full understanding of the model should be developed, examining many different classes of compounds before its full commercial use.

#### 3.3.1. Corrosion Test

**Figure 5:**
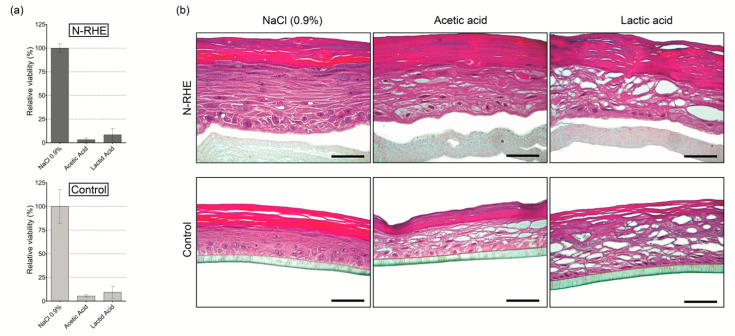
In vitro evaluation of skin corrosion of N-RHE and epidermal control (USP-RHE): (**a**) Viability, according to OECD TG 431. Results are expressed as mean ± standard deviation of two independent experiments (n = 2), each tested with three different tissue replicates; (**b**) Histological analysis of H&E stained vertical paraffin sections. Results were obtained after exposure to substances: NaCl (0.9%), acetic acid, and lactic acid. Bar = 50 µm.

This test’s importance was to determine the N-RHE model’s predictive capacity to be used for in vitro skin corrosion testing performed according to OECD TG 431 [46]. The potential of the N-RHE model was reflected in the RV values and morphological characteristics after the topical application of chemical substances known for their corrosion properties on the skin. Three substances were evaluated: sodium chloride (0.9%), acetic acid, and lactic acid. In the initial stage, three tissues of the N-RHE model were incubated with the mentioned substances for 60 min, as detailed in Section 2.7.1. Similar behavior of RV values was observed after chemical treatment (Figure 5a) of the N-RHE model compared to the control model (USP-RHE). The treatment with acetic acid produced RV values of 3.7% and 5.7%, whereas lactic acid yielded 10.9% and 8.8% for N-RHE and control models, respectively. Similar results of RV were described for the commercial EpisSkin model [66] (4.8% for acetic acid and 8.2% for lactic acid).

After chemical treatment, tissue damage was accompanied by morphological evaluations of the histological images (Figure 5b). The N-RHE model’s morphology presents alterations due to exposition to corrosive substances (acetic acid and lactic acid). The images show evidence of non-adhesion between cells in the epidermis’ intermediate strata (vacuolization) compared to the models exposed with PBS (non-corrosive substance) because the latter is considered the negative control.

Hence, the applicability of the N-RHE model as a platform for skin corrosion testing is demonstrated. From our results, it was possible to find a similarity between our model when compared to EpiSkin^TM^, EpiDerm^TM^ SCT, and SkinEthic^TM^ RHE [13].

#### 3.3.2. Irritation Test

**Figure 6:**
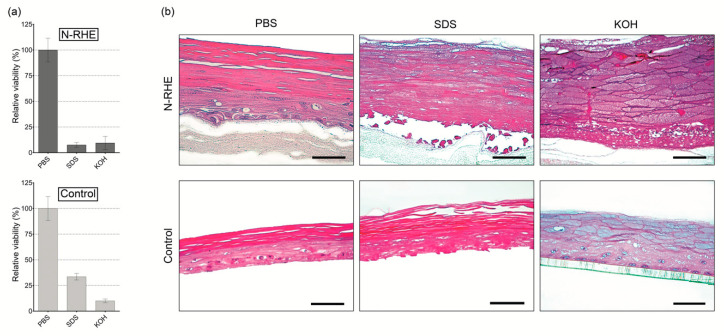
In vitro evaluation of skin irritation of N-RHE and control (USP-RHE) epidermal models: (**a**) relative cell viability testing, according to OECD TG 439. Results are expressed as mean ± standard deviation of two independent experiments (n = 2), each tested with three different tissue replicates; (**b**) Histological analysis of H&E stained vertical paraffin sections. Results were obtained after exposure to substances: PBS, SDS (5%), and KOH (5%). Bar = 50 µm.

Among the chemical substances suggested by OECD TG439 [45], PBS (negative control), SDS (irritant), and KOH (irritant) were selected. The RV value of each testing substance was calculated relative to the RV value of the negative control, established as 100%. PBS and SDS, as negative and positive controls, respectively, are classically used as references in skin irritation testing [67,68]. As established in OECD TG439 [45], if the tissue RV value obtained after exposure to chemicals is less than or equal to 50%, a model is considered to have reacted to the damage of an irritating product [45]. This characteristic was observed in the behavior of our model for SDS (RV between 6% and 10%) and KOH (RV between 4% and 21%) (Figure 6a).

The results obtained in this applicability evaluation of the N-RHE model for the irritation test were according to those obtained in the control model, as attested by RV evaluation (Figure 6a) and morphological characterization (Figure 6b). Figure 6a shows RV values for the N-RHE model, similar to the control model. Moreover, the obtained results are comparable to those described by the United Nations Globally Harmonized System (UN GHS) in their classification of irritating substances [45] (Table S1). Thus, we may determine the similarity of value of RV(SDS 5%) and standard deviation (SD) of the commercial epidermal models [45] EpiSkin^TM^ (RV < 40%, SD ≤ 18%), EpiDerm^TM^ SIT (RV < 20%, SD ≤ 18%), SkinEthic RHE^TM^ (RV < 40%, SD ≤ 18%) and LabCyte EPI-MODWL24 SIT (RV < 40%, SD ≤ 18%) with our model N-RHE.

The tissue structural organization assessment by histological analysis of both RHEs models shows that the negative controls for irritation (Figure 6b) are comparable to those exposed in Figure 4a, corroborating the non-damaging action of PBS. In contrast, the morphology of the tissues treated with SDS and KOH (Figure 6b) revealed a high degree of damage, from the outermost part (stratum corneum) to the stratum basal, as a response to the inflammatory process. Skin irritations are complex biological processes in which acute reactions are seen after immediate contact with chronic dermatitis [45,69]. On the other hand, “irritants” are substances that have reversible effects on the skin [69]. These results are directly related to the values of RV < 50%, characteristic of irritating substances.

These first results (RV and histology) indicate that our model is a promising platform to evaluate skin irritation. However, more chemical substances must be tested, as detailed in the list provided by OECD TG439 [45].

## 4. Conclusions

Our study demonstrated the obtainment of in vitro constructions of epidermal equivalent by using porous electrospun mats without collagen. By avoiding animal protein use, we seek to increase the model’s predictive capacity as a response to a human tissue model. Three varieties of fibrous scaffolds have been constructed using synthetic polymers (PET, PBT, N6/6). It was possible to demonstrate morphologically the presence of epithelial tissue in the FTSs construction process. In the construction of the epidermis in vitro in N6/6, the stratification of keratinocytes was successfully achieved. The obtained results reveal a promising platform for irritation and corrosion tests.

The novelty presented here combines a well-known scaffold production technique and a successful skin model protocol, followed by its validation. Moreover, this model is based on collagen’s exemption, the use of inexpensive commodity plastics, flexibility, and readiness to build by the use of porous electrospun mats, properties that can facilitate production and increase its availability.

## Figures and Tables

**Table 1:** Percentage of polymers used in the preparation of polymer solutions in different proportions in solvent mixtures.

Solution Concentration (*w/v*) %	Solvent Mixture (*v/v*)
PET (20% and 30%)	HFP:DCM (10:0, 7:3 and 1:1)
PBT (20% and 30%)	HFP:DCM (10:0, 7:3 and 1:1)
N6/6 (12.5%)	FAc:CHCl_3_ (7.5:2.5)

**Table 2:** Parameters ^1^ established in the electrospinning process of PET, PBT, and N6/6.

Polymer	Process Parameters		
	Voltage (kV)	Flow Rate (mL·h^−1^)	Tip-to-Collector Distance (cm)
PET	20	12	30
PBT	20	12	30
N6/6	20	2	19

^1^ Shared parameters: 54% (±2%) humidity, 19 °C (±2 °C) temperature, 20 gauge needle as a spinneret.

## Supplementary Materials

The following are available online at https://www.mdpi.com/2079-4991/10/12/2528/s1, Figure S1. SEM analysis for electrospinning different PET solutions, Figure S2. SEM analysis for electrospinning different PBT solutions, Figure S3. Histogram and normal probability plot for fiber diameter analysis of PET electrospun mats, Figure S4. Histogram and normal probability plot for fiber diameter analysis of PBT electrospun mats, Figure S5. Box plot the distribution of fiber diameters analysis of PET electrospun mats, Figure S6. Box plot the distribution of fiber diameters analysis of PBT electrospun mats, Figure S7. Box plot the distribution of fiber diameters analysis of N6/6 electrospun mats, Figure S8: Stained human skin and RHE sections, Figure S9. Western blot analysis of the RHE samples: N-RHE and Control, Table S1. Comparison between the UN GHS category for skin irritation, N-RHE model, and control model according to OECD TG 439.
