# Supplementary material for: Development of Epidermal Equivalent from Electrospun Synthetic Polymers for In Vitro Irritation/Corrosion Testing
Source: Nanomaterials (Basel). 2020 Dec 16;10(12):2528. doi: 10.3390/nano10122528 (PMC7766501; doi:10.3390/nano10122528)
Supplement: Supplementary file 1 [file nanomaterials-10-02528-s001.pdf]

nanomaterials

MDPI

Supplementary Material

DEVELOPMENT OF EPIDERMAL EQUIVALENT FROM ELECTROSPUN SYNTHETIC POLYMERS FOR IN VITRO IRRITATION/CORROSION TESTING

Denisse Esther Mallaupoma Camarena [1], Larissa Satiko Alcântara Sekimoto Matsuyama [2], Silvya Stuchi Maria-Engler [2] and Luiz Henrique Catalani [1,*]

[1] Laboratory of Polymeric Biomaterials, Department of Fundamental Chemistry, Institute of Chemistry, University of São Paulo, 05508-000, São Paulo, Brazil; denisse23uni@usp.br (D.E.M.C); catalani@usp.br (L.H.C)

[2] Skin Biology Laboratory, Clinical Chemistry & Toxicology Department, School of Pharmaceutical Sciences, University of São Paulo, 05508-000, São Paulo, Brazil; silvya@usp.br (S.S.M-E)

* **Correspondence:** catalani@usp.br; Tel.: +55-11-97272-8877 (L.H.C.)

Figure S1. SEM analysis for electrospinning different PET solutions

SEM micrographs showing the morphology of mats obtained by electrospinning of (a) 20% PET and (b) 30% PET, using three different solvent mixtures of HFP/DCM (20 kV Applied Voltage, Flow Rate 12mL/h). Proportion of HFP/DCM (10:0), HFP/DCM (7:3) and HFP/DCM (1:1). The conditions that produced mats with beads were discarded. Yellow arrows point to beads. Dichloromethane and chloroform are exchangeable as a solvent constituent.

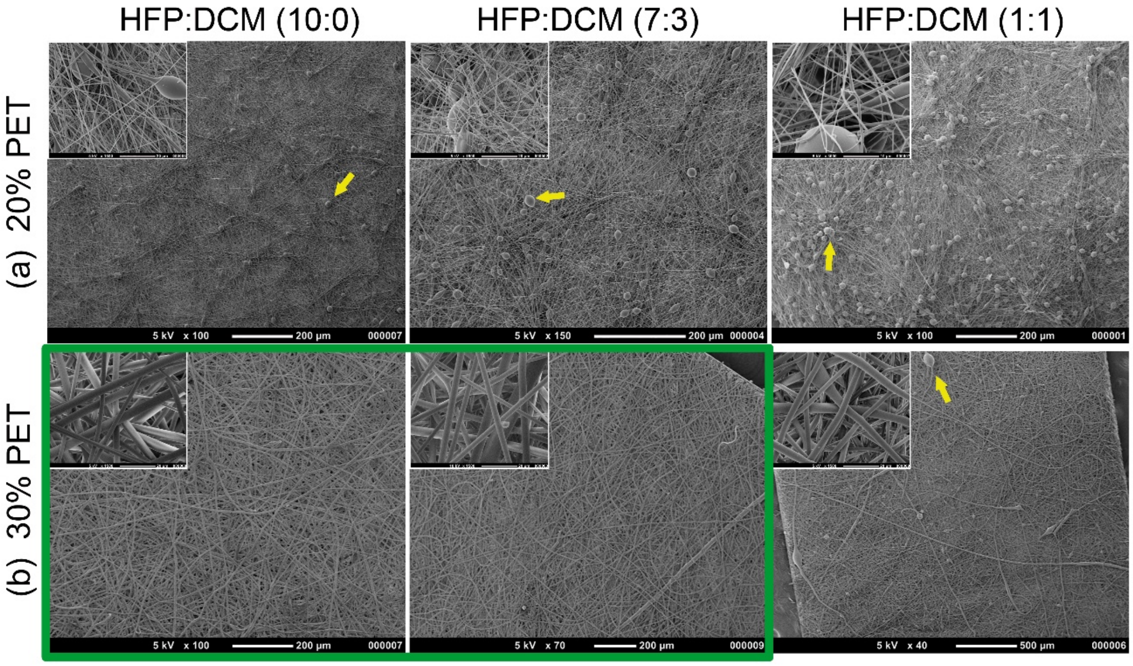

Green box show images from the conditions that generated bead-free mats

Figure S2. SEM analysis for electrospinning different PBT solutions

SEM micrographs showing the morphology of mats obtained by electrospinning of (a) 10% PBT and (b) 20% PBT, using three different solvent mixtures of HFP/DCM (20 kV Applied Voltage, Flow Rate 12mL/h). Proportion of HFP/DCM (10:0), HFP/DCM (7:3) and HFP/DCM (1:1). Yellow arrows point to beads. Dichloromethane and chloroform are exchangeable as a solvent constituent. Mats from 20% PBT (HFP:DCM - 1:1) showed adhesion problems to remove from the collector.

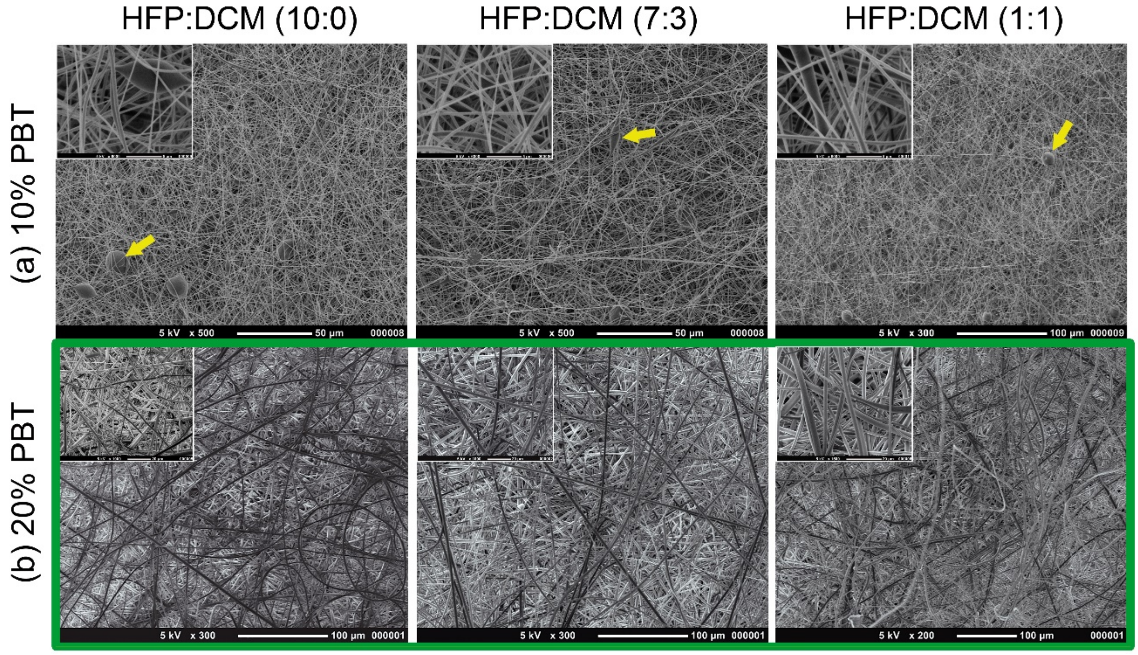

Green box shows images from the conditions that generated bead-free mats.

Figure S3. Histogram and normal probability plot for fiber diameter analysis of PET electrospun mats

Hypothesis test to confirm the normality of the data distribution (fiber diameters) of PET (fibers were bead-free). The electrospinning parameters used were: 30% PET concentration, 20 kV voltage, 30 cm distance between needle and collector, 12 mL/h flow rate. Proportion of HFP/DMC (10:0), HFP/DMC (7:3) and HFP/CHCl3 (7:3). Total of 400 data per condition (a,b,c,d).

If p-value> 0.05, the normal hypothesis is accepted. Then the histogram can be represented by the Gaussian curve. From the data a, b, c, d the p <0.05 were obtained, it is concluded that the data do not present a normal distribution.

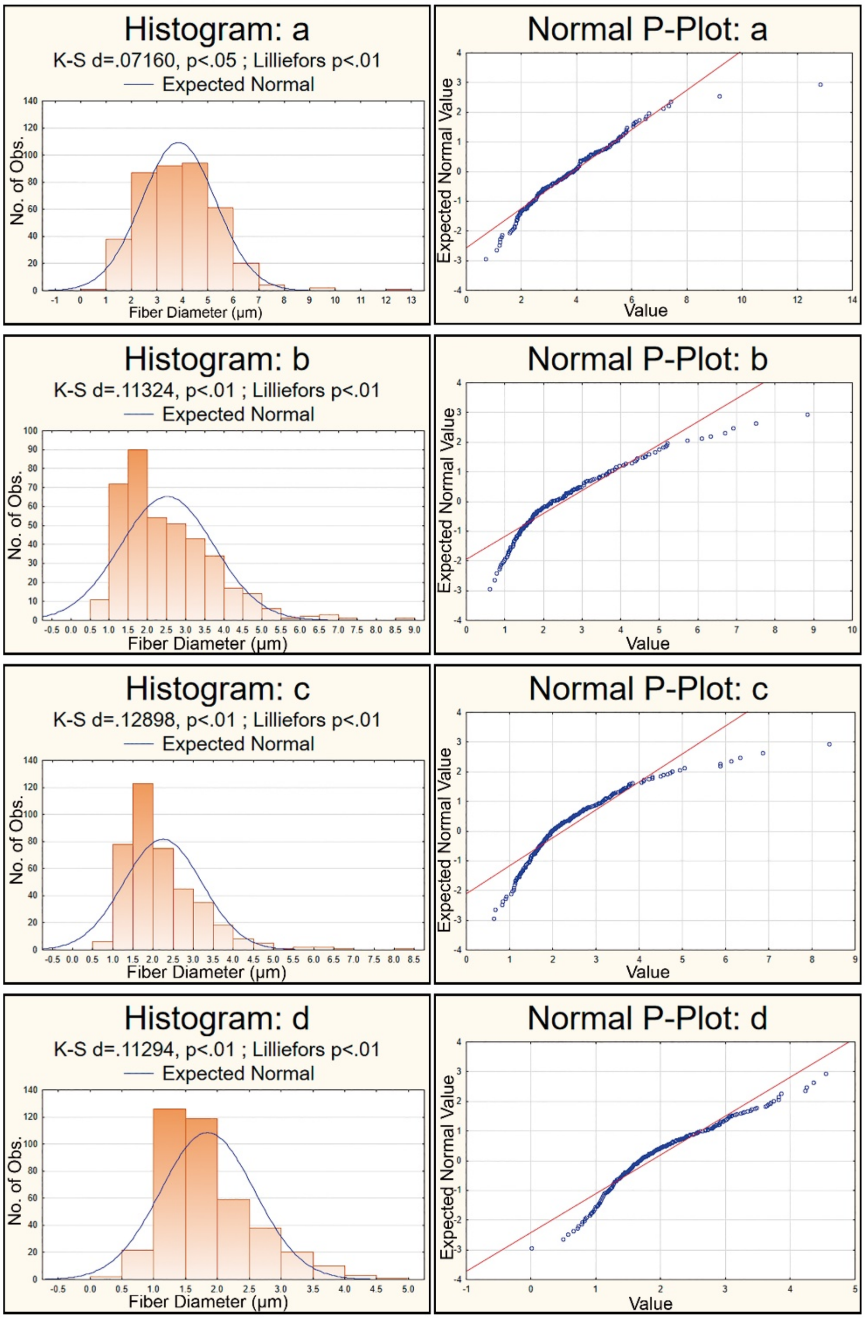

[a] 30% PET, HFP:DCM (10:0)

[b,c] Repetitions of 30% PET, HFP:DCM (7:3)

[d] 30% PET, HFP: $CHCl_3$ (7:3)

Figure S4. Histogram and normal probability plot for fiber diameter analysis of PBT electrospun mats

Hypothesis test to confirm the normality of the data distribution (fiber diameters) of PBT (fibers were bead-free). The electrospinning parameters used were: 20% PBT concentration, 20 kV voltage, 30 cm distance between needle and collector, 12 mL/h flow rate. Proportion of HFP/DMC (10:0), HFP/DMC (7:3) and HFP/CHCl3 (7:3). Total of 400 data per condition (a,b,c,d).

If p-value> 0.05, the normal hypothesis is accepted. Then the histogram can be represented by the Gaussian curve. From the data a, b, c, d the p <0.05 were obtained, it is concluded that the data do not present a normal distribution.

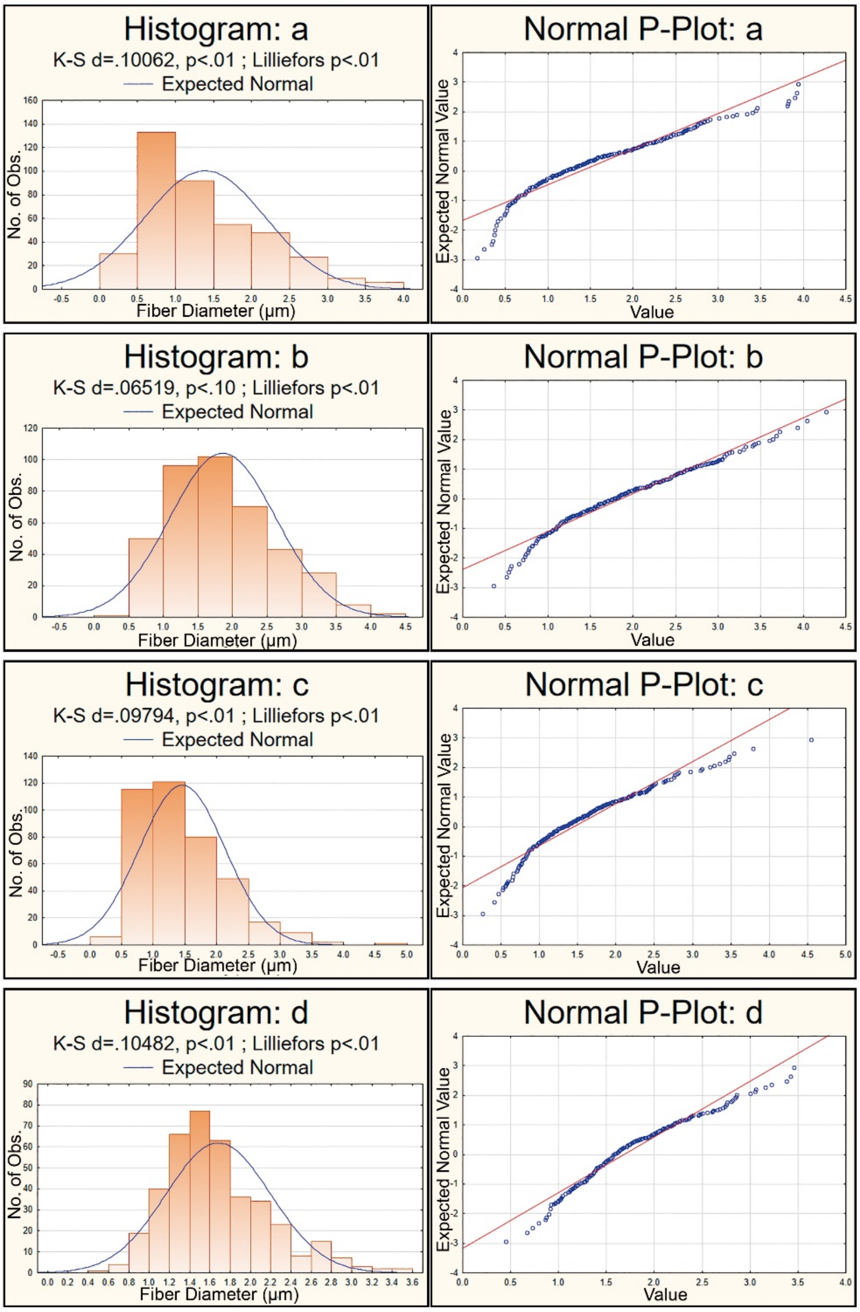

[a] 20% PBT, HFP:DCM (10:0)

[b,c] Repetitions of 20% PBT, HFP:DCM (7:3)

[d] 20% PBT, HFP: $CHCl_3$ (7:3)

Figure S5. Box plot the distribution of fiber diameters analysis of PET electrospun mats

The electrospinning parameters used were: 30% PET concentration, 20 kV voltage, 30 cm distance between needle and collector, 12 mL/h flow rate. Box plot analyses refers to solvent mixtures HFP/DMC: 10:0 (a), HFP/DMC 7:3 (b and c) and HFP/$CHCl_3$ 7:3 (d). Total of 400 data per condition (a, b, c, d).

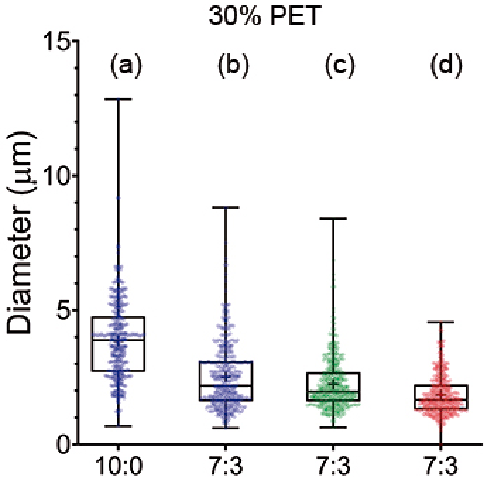

Statistics data	Boxplot of 30% PET			
	a	b	c	d
P_{25}	2.71	1.62	1.61	1.31
P_{50}	3.88	2.19	1.97	1.66
P_{75}	4.76	3.09	2.68	2.23
IQR	2.05	1.47	1.07	0.92
Mean+	3.85	2.52	2.24	1.85
SD	1.46	1.22	0.98	0.74

[a] 30% PET, HFP:DCM (10:0)

[b,c] Repetitions of 30% PET, HFP:DCM (7:3)

[d] 30% PET, HFP: $CHCl_3$ (7:3)

[+] Mean

P_{25}: 25% percentile, P_{50}: 50% percentile (median), P_{75}: 75% percentile, IQR: Interquartile range, IQR= P_{75} – P_{25}, SD: standard deviation.

Figure S6. Box plot the distribution of fiber diameters analysis of PBT electrospun mats

The electrospinning parameters used were: 20% PBT concentration, 20 kV voltage, 30 cm distance between needle and collector, 12 mL/h flow rate. Box plot analyses refers to solvent mixtures HFP/DMC: 10:0 (a), HFP/DMC 7:3 (b and c) and HFP/CHCl3 7:3 (d). Total of 400 data per condition (a, b, c, d).

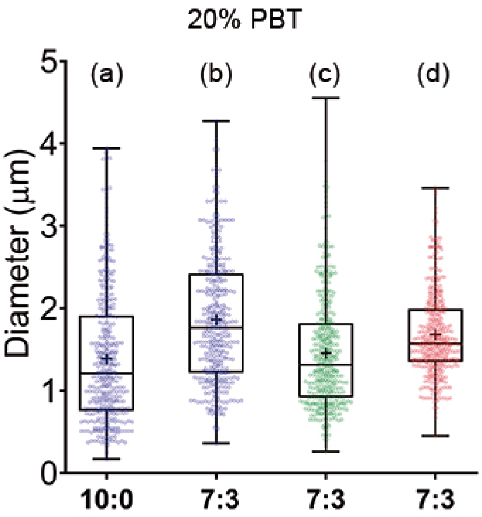

Statistics data	Boxplot of 20% PBT			
	a	b	c	d
P_{25}	0.76	1.22	0.92	1.35
P_{50}	1.21	1.77	1.32	1.57
P_{75}	1.91	2.42	1.82	1.99
IQR	1.15	1.20	0.90	0.64
Mean⁺	1.39	1.86	1.46	1.68
SD	0.80	0.77	0.67	0.52

[a] 20% PBT, HFP:DCM (10:0)

[b,c] Repetitions of 20% PBT, HFP:DCM (7:3)

[d] 20% PBT, HFP: $CHCl_3$ (7:3)

[+] Mean

P_{25}: 25% percentile, P_{50}: 50% percentile (median), P_{75}: 75% percentile, IQR: Interquartile range, IQR= P_{75} – P_{25}, SD: standard deviation.

Figure S7. Box plot the distribution of fiber diameters analysis of N6/6 electrospun mats

Repetitions of Electrospinning of the N6/6 solutions (12 %w/v) in AF/CHCl3 (7.5: 2:5), was carried out using the following conditions:20 kV applied voltage, flow rate 2mL/h and 19 cm of tip to collector distance. The polymer fibers were collected over static collector. Total of 400 data per repetition (a, b, c).

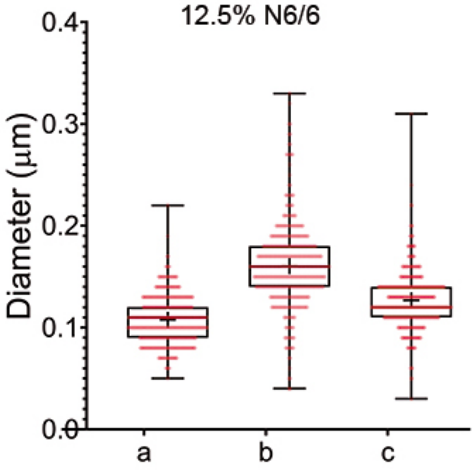

Statistics data	Boxplot of 12.5% N6/6		
	a	b	c
P_{25}	0.09	0.14	0.11
P_{50}	0.11	0.16	0.12
P_{75}	0.12	0.18	0.14
IQR	0.03	0.04	0.03
Mean+	0.11	0.16	0.13
SD	0.02	0.04	0.03

[a,b,c] Repetitions of 12.5% N6/6

P_{25}: 25% percentile, P_{50}: 50% percentile (median), P_{75}: 75% percentile, IQR: Interquartile range, IQR= $P_{75} - P_{25}$, SD: standard deviation.

Figure S8: Stained human skin and RHE sections

Histology and immunohistochemistry of human skin and RHE model reported by Pedrosa et al (with permission from the publisher) [1].

Histological analysis of hematoxylin/eosin stained vertical paraffin sections of (A) native human epidermis and (B) USP-RHE model. USP-RHE model is presenting all strata, e.g, basale, spinosum, granulosum and corneum. (C) Immunofluorescent staining of USP-RHE model (Day 12) and human epidermis with cytokeratin 10, with negative marker of CK10 in the basal layer (CK10). Cytokeratin 14 (CK14) expression is under normal condition confined to the basal cell layer of the epidermis. Magnification =20 X. Bar =200 μm.

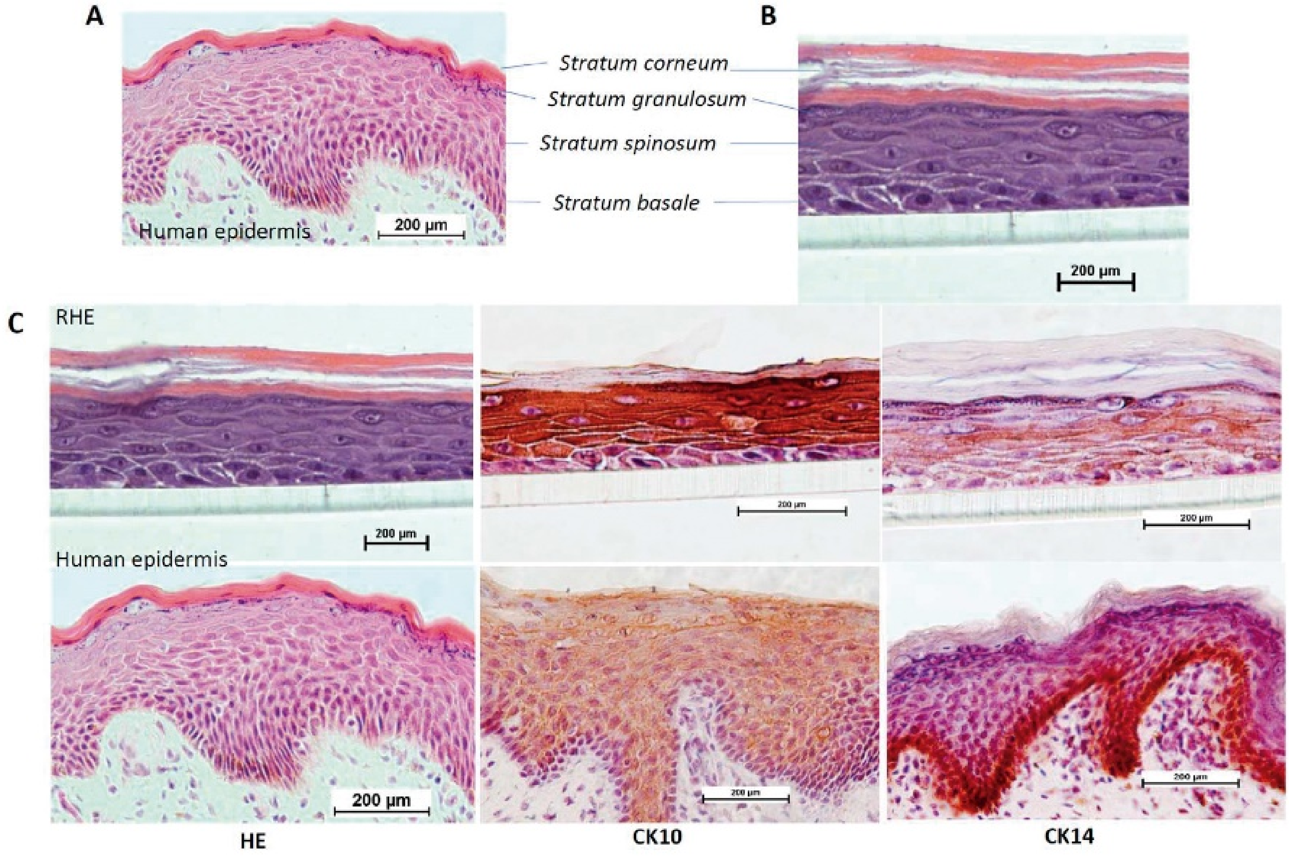

Figure S9. Western blot analysis of the RHE samples: N-RHE and Control

Western Blotting membranes after revealing the antibodies and confirm the presence of the following proteins: β-ACT, KRT14, KRT10 and IVL in Control and N-RHE. Uncut images. __ N-RHE is the RHE model with N6/6 scaffold, and Control is the USP-RHE model

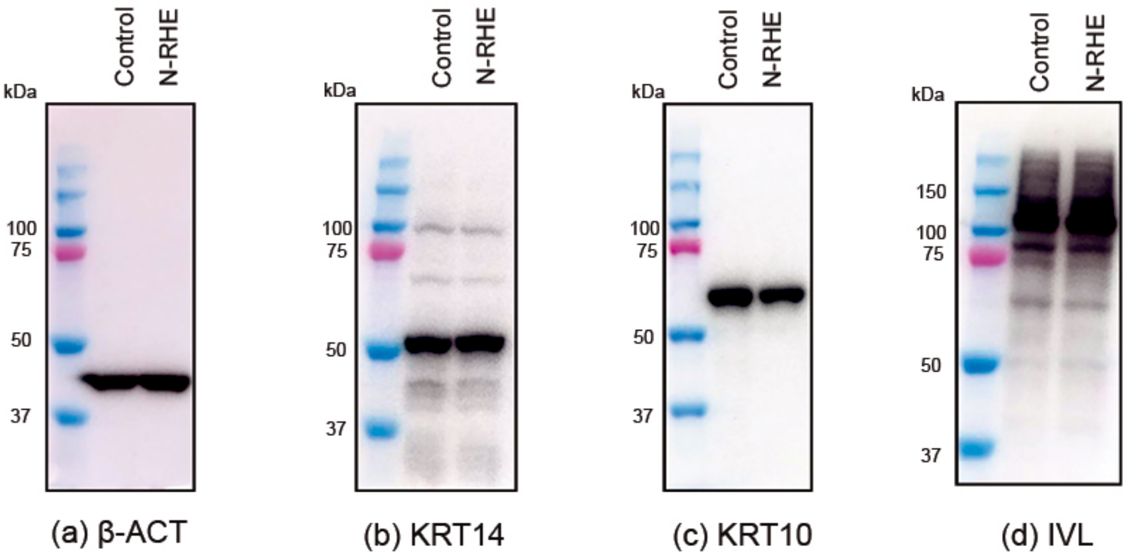

Table S1. Comparison between the UN GHS category for skin irritation, N-RHE model, and control model according to OECD TG 439.

Substance	Experiment[a]	$RV_{N\text{-}RHE}$ (%) Mean ± SD	N-RHE model	$RV_{control}$ (%) Mean ± SD	Control model	UN GHS Cat.
PBS		100		100		
SDS (5%)	1	5.75 ± 2.27	I	36.55 ± 0.76	I	I
	2	10.27 ± 1.75	I	30.60 ± 0.71	I	
KOH (5%)	1	3.96 ± 0.61	I	9.07 ± 0.89	I	I
	2	20.56 ± 2.07	I	11.50 ± 1.96	I	

UN GHS= United Nations Globally Harmonized System; SD = standard deviation; I= irritant

[a] independent experiments, in each experimental triplicate

References

[1] This figure was published in Pedrosa, T. do N.; Catarino, C. M.; Pennacchi, P. C.; Assis, S. R. de; Gimenes, F.; Consolaro, M. E. L.; Barros, S. B. de M.; Maria-Engler, S. S. A New Reconstructed Human Epidermis for in Vitro Skin Irritation Testing. *Toxicol. Vitr.*, **2017**, *42*, 31–37. https://doi.org/10.1016/j.tiv.2017.03.010. Copyright Elvesier, 2017.
